# cfMethDB: A Comprehensive cfDNA Methylation Data Resource for Cancer Biomarkers

**DOI:** 10.1093/gpbjnl/qzaf092

**Published:** 2025-10-14

**Authors:** Yuanhui Sun (孙元辉), Zhixian Zhu (朱志贤), Qiangwei Zhou (周强伟), Zhe Wang (王哲), Yuying Hou (侯钰莹), Xionghui Zhou (周雄辉), Guoliang Li (李国亮)

**Affiliations:** National Key Laboratory of Crop Genetic Improvement, Hubei Hongshan Laboratory, Huazhong Agricultural University, Wuhan 430070, China; Agricultural Bioinformatics Key Laboratory of Hubei Province, Hubei Engineering Technology Research Center of Agricultural Big Data, Key Laboratory of Smart Farming Technology for Agricultural Animals, 3D Genomics Research Center, College of Informatics, Huazhong Agricultural University, Wuhan 430070, China; National Key Laboratory of Crop Genetic Improvement, Hubei Hongshan Laboratory, Huazhong Agricultural University, Wuhan 430070, China; Agricultural Bioinformatics Key Laboratory of Hubei Province, Hubei Engineering Technology Research Center of Agricultural Big Data, Key Laboratory of Smart Farming Technology for Agricultural Animals, 3D Genomics Research Center, College of Informatics, Huazhong Agricultural University, Wuhan 430070, China; National Key Laboratory of Crop Genetic Improvement, Hubei Hongshan Laboratory, Huazhong Agricultural University, Wuhan 430070, China; Agricultural Bioinformatics Key Laboratory of Hubei Province, Hubei Engineering Technology Research Center of Agricultural Big Data, Key Laboratory of Smart Farming Technology for Agricultural Animals, 3D Genomics Research Center, College of Informatics, Huazhong Agricultural University, Wuhan 430070, China; National Key Laboratory of Crop Genetic Improvement, Hubei Hongshan Laboratory, Huazhong Agricultural University, Wuhan 430070, China; Agricultural Bioinformatics Key Laboratory of Hubei Province, Hubei Engineering Technology Research Center of Agricultural Big Data, Key Laboratory of Smart Farming Technology for Agricultural Animals, 3D Genomics Research Center, College of Informatics, Huazhong Agricultural University, Wuhan 430070, China; National Key Laboratory of Crop Genetic Improvement, Hubei Hongshan Laboratory, Huazhong Agricultural University, Wuhan 430070, China; Agricultural Bioinformatics Key Laboratory of Hubei Province, Hubei Engineering Technology Research Center of Agricultural Big Data, Key Laboratory of Smart Farming Technology for Agricultural Animals, 3D Genomics Research Center, College of Informatics, Huazhong Agricultural University, Wuhan 430070, China; National Key Laboratory of Crop Genetic Improvement, Hubei Hongshan Laboratory, Huazhong Agricultural University, Wuhan 430070, China; Agricultural Bioinformatics Key Laboratory of Hubei Province, Hubei Engineering Technology Research Center of Agricultural Big Data, Key Laboratory of Smart Farming Technology for Agricultural Animals, 3D Genomics Research Center, College of Informatics, Huazhong Agricultural University, Wuhan 430070, China; National Key Laboratory of Crop Genetic Improvement, Hubei Hongshan Laboratory, Huazhong Agricultural University, Wuhan 430070, China; Agricultural Bioinformatics Key Laboratory of Hubei Province, Hubei Engineering Technology Research Center of Agricultural Big Data, Key Laboratory of Smart Farming Technology for Agricultural Animals, 3D Genomics Research Center, College of Informatics, Huazhong Agricultural University, Wuhan 430070, China

**Keywords:** Database, cfDNA, DNA methylation, Pan-cancer, Biomarker

## Abstract

Cancer is a major global health threat, and early detection is crucial for improving patient outcomes. DNA methylation in circulating cell-free DNA (cfDNA) has emerged as a promising biomarker for non-invasive cancer diagnosis. However, the integration and utilization of existing cfDNA methylation data have been limited, hindering comprehensive research efforts, particularly in the discovery of cfDNA methylation biomarkers. To address this challenge, we introduced cfMethDB, a comprehensive database dedicated to cfDNA methylation in cancer that encompasses 4828 publicly available datasets. Through standardized analysis, we identified 1,048,770 differentially methylated cytosines (DMCs) as candidate biomarkers across seven cancer types. With cfMethDB, we not only identified known cfDNA methylation biomarkers, but also discovered several genes, such as *ZIC4*, that could be novel biomarkers. Moreover, cfMethDB offers a suite of user-friendly tools, including biomarker evaluation, pan-cancer search, and end motif analysis. We hope that cfMethDB will serve as a valuable platform for the discovery of novel cancer cfDNA methylation biomarkers and facilitate cancer research and clinical applications. cfMethDB is publicly available at https://cfmethdb.hzau.edu.cn/home.

## Graphical abstract

**Figure:**
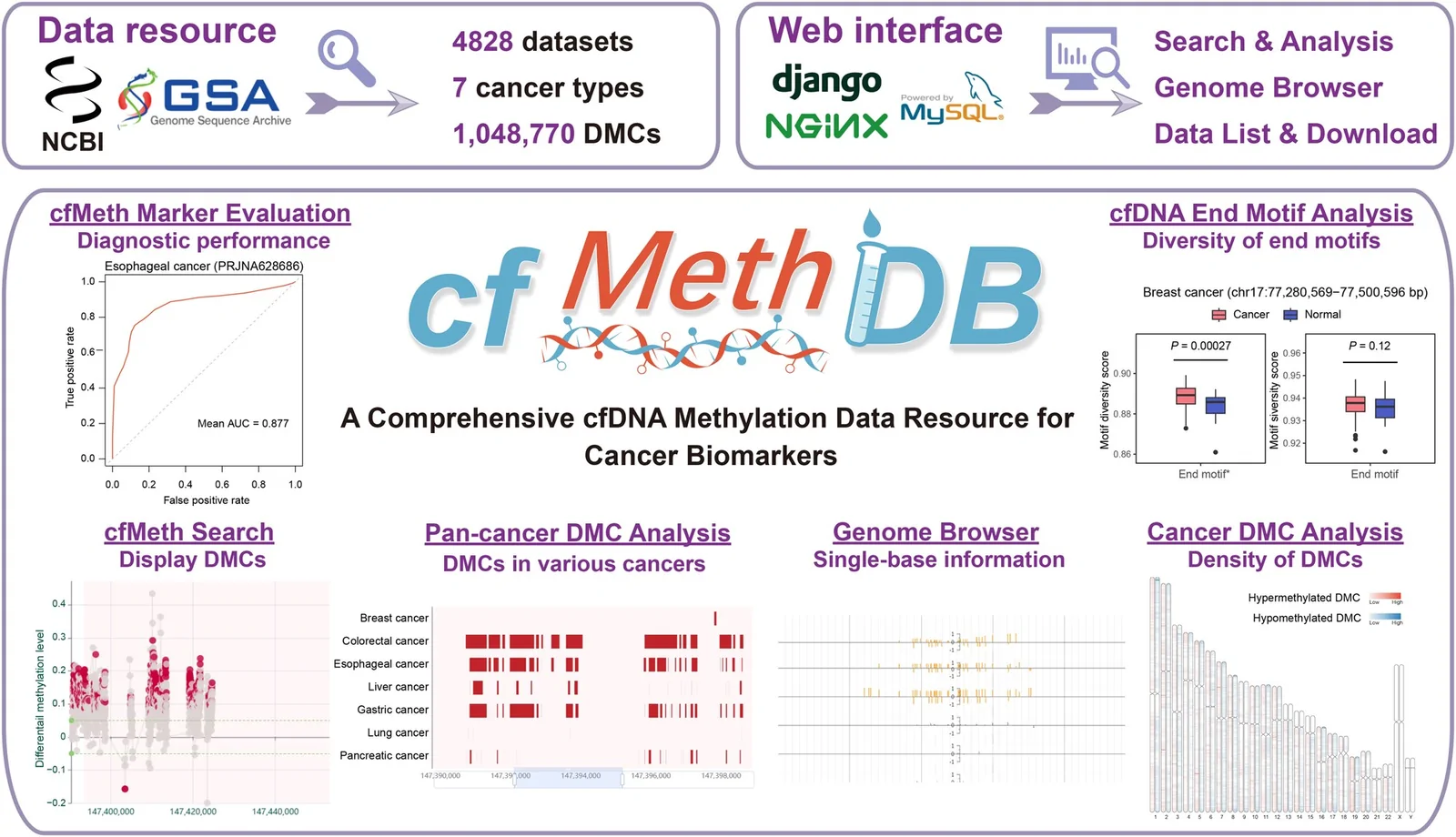

## Introduction

Cancer is a major threat to human health, with nearly 20 million new cases and 9.7 million deaths reported worldwide in 2022 [1]. Early detection of cancer is a viable strategy for improving outcomes in terms of population health [2]. For instance, delays in breast cancer treatment, particularly those exceeding three months, are associated with advanced-stage diagnosis and reduced survival rates [3]. In the THUNDER study, the interception model projected that, compared to usual care, 38.7% to 46.4% of patients who were diagnosed at late stages could be identified at earlier stages using the early diagnosis method [4].

For early cancer diagnosis, minimally invasive sampling methods, such as collecting cell-free DNA (cfDNA) from the plasma, are preferred [5]. cfDNA is released by dead cells into the bloodstream [6], offering a new method for non-invasive cancer diagnosis [7]. Many cancer-associated molecular features have been detected in the cfDNA of patients with various cancer types [8]. Among those features, cfDNA methylation has emerged as the most promising signal for cancer detection [2]. Recently, many studies have utilized cfDNA methylation signals for the early diagnosis of various cancers. For example, the methylation of *SEPT9* can be used to detect the majority of colorectal cancer patients at all stages [9]. Similarly, the combined methylation of *HOXA9* and *HIC1* serves as an effective diagnostic biomarker for early ovarian cancer screening [10]. Methylation of the *GSTP1* promoter is highly specific for diagnosing prostate cancer [11]. Additionally, many methylation biomarkers have been identified in other cancer types, including lung cancer [12], liver cancer [13], and breast cancer [14]. With the rapid development of cfDNA methylation-based diagnosis, a vast amount of cfDNA methylation data has been generated. Although some databases contain cfDNA methylation data, effectively integrating and utilizing these data remains challenging. The Cell-Free Epigenome Atlas (CFEA) database encompasses three widely recognized epigenetic modifications on cfDNA; however, its cfDNA methylation data are limited and have not been updated recently [15]. cfOmics provides multi-omics liquid biopsy data, including cfDNA and cfRNA; however, this database does not primarily focus on cfDNA methylation data, and lacks DNA methylation information at single-base resolution [16]. These limitations hinder the comprehensive exploration of cfDNA methylation across the whole genome and particularly affect the discovery of cfDNA methylation biomarkers for cancer diagnosis. In addition, the characteristics of cfDNA fragments, such as fragment sizes and end motifs, are crucial for cfDNA analysis [17,18]. Incorporating these fragment features into the cfDNA database is essential for obtaining a deeper understanding of cfDNA.

In this study, we introduced cfMethDB (https://cfmethdb.hzau.edu.cn/home), a comprehensive cfDNA methylation data resource for cancer biomarkers, which includes 4828 publicly available cfDNA methylation datasets. Using a standardized analysis pipeline, we identified 1,048,770 differentially methylated cytosines (DMCs) from cfDNA methylation data as candidate biomarkers across 7 types of cancer. With cfMethDB, users can explore these biomarkers across the entire genomic landscape rather than being limited to specific regions, such as genes or promoters. cfMethDB provides detailed information on DMCs and offers a wide range of functions, including region search, pan-cancer DMC search, and a diagnostic evaluation tool, to help researchers explore candidate methylation biomarkers. Moreover, cfMethDB offers information on the fragmentation features of cfDNA, assisting researchers in exploring the intrinsic properties of cfDNA.

To our knowledge, cfMethDB is the first database to provide cancer cfDNA methylation biomarkers with precise genomic location information. It enables researchers to easily explore the differences in cfDNA methylation in cancer, facilitating clinical diagnosis and treatment.

## Data collection and processing

### Collection and analysis of cfDNA methylation data

**Figure 1:**
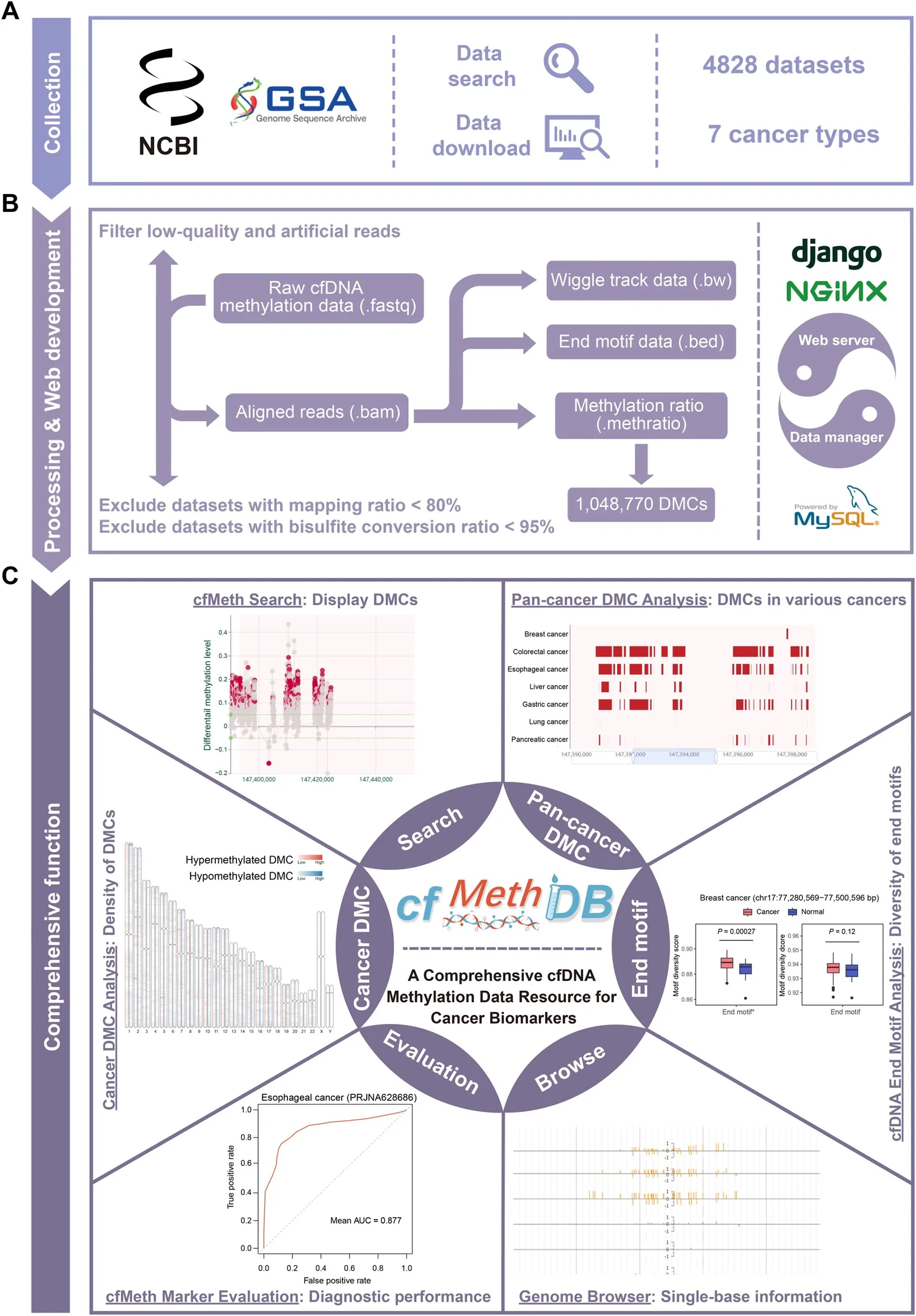
Workflow of the cfMethDB database **A**. Collection of cfDNA methylation datasets from the NCBI SRA and GSA databases. **B**. Data processing and web development for cfMethDB. **C**. Comprehensive functional and analysis modules in cfMethDB. cfDNA, cell-free DNA; NCBI, National Center for Biotechnology Information; SRA, Sequence Read Archive; GSA, Genome Sequence Archive; DMC, differentially methylated cytosine.

We collected cfDNA methylation data from the National Center for Biotechnology Information (NCBI) Sequence Read Archive (SRA) database [19] and the Genome Sequence Archive (GSA) database [20] up to June 2024. A total of 7530 datasets were downloaded initially (**Figure 1**A).

The collected cfDNA methylation datasets were generated using various methods, including whole-genome bisulfite sequencing (WGBS), reduced representation bisulfite sequencing (RRBS), and padlock-based methods. The primary difference in data analysis among these methods was in the preprocessing stage. Fastp [21] was used to trim low-quality and artificial reads with default parameters. Specifically, Trim Galore with the “*-rrbs*” parameter was used to preprocess RRBS data. Trim Galore detects sequences affected by adapter contamination and removes an additional 2 bp from the end of reads to account for technical biases introduced by the RRBS technique [22]. BatMeth2 [23] was subsequently used to map the trimmed reads to the human reference genome (hg38), and the mapping ratio was calculated using SAMtools [24]. Datasets with a mapping ratio below 80% were filtered out. After mapping, reads with an alignment quality score below 30 and a coverage of each cytosine less than 2 were discarded. The “Calmeth” module in BatMeth2 was used to calculate the DNA methylation level of each cytosine (Figure 1B). The bisulfite conversion rate was evaluated by calculating the CHG methylation level as described previously [25], and datasets with a conversion rate lower than 95% were excluded from downstream analysis. Finally, 4828 datasets were retained for further analysis, and the number of CpG sites per sample is displayed in Figure S1A.

### Collection of biomarker genes from literature

Initially, we retrieved abstracts of published literature from PubMed and extracted sentences related to DNA methylation, various cancer types, and genes. After manual curation, we compiled a total of 2611 biomarker records, including 87 DNA methylation biomarker genes in cfDNA and 697 genes in tissue. In total, cfMethDB contains 729 unique genes in the curated biomarker records. We utilized word cloud plots and tables for intuitive visualization of these biomarker records.

### DMC analysis

To identify DMCs across the whole genome for each cancer type, we used SMART2 [26] in the DMC mode with default parameters (Figure 1B). DMCs with an absolute methylation difference greater than 0.05 and a *P* value less than 0.01 were retained for downstream analysis. The ChIPseeker R package [27] was used to annotate these DMCs. The DMCs included in cfMethDB covered most of the known DNA methylation biomarkers, including approximately 94% (686 of 729) of those previously reported in the literature and all 37 biomarkers approved by the National Medical Products Administration (NMPA) (Table S1).

### cfDNA fragmentomics analysis

cfDNA fragmentation patterns are promising biomarkers for cancer detection. For example, the frequency of end motifs can effectively distinguish cancer samples from normal samples [17]. In this study, cfDNA fragment sizes were calculated using the “CollectInsertSizeMetrics” module in GATK [28]. The distribution of cfDNA fragment lengths across different projects is illustrated in Figure S1B. For end motifs, we used BEDTools [29] to extract the first four bases from the 5′-ends of the fragments. We also introduced the character ‘U’ to indicate unmethylated cytosines to distinguish the methylation status of the cytosines. The frequency of each end motif was calculated by dividing the number of occurrences of that motif by the total number of reads in the region. The motif diversity score was computed using the methodology outlined previously [17].

### Diagnostic analysis of methylation biomarkers

In cfMethDB, users can select the cancer type and project of interest, and extract DNA methylation levels from the queried regions. Any queried regions with missing values more than 20% were excluded from further analysis. For the remaining queried regions, missing values were imputed using the “na_interpolation” function of the imputeTS R package [30], which employs a linear interpolation method. Then, a logistic regression-based diagnostic model was constructed with the caret R package [31] on the whole dataset of the selected project, with five-fold cross-validation repeated 20 times.

### Database implementation

cfMethDB offers several search and analysis functions complemented by a genome browser powered by JBrowse [32]. The data within cfMethDB were stored in MySQL, and the web interface was built using Nginx and the Django framework, providing a user-friendly experience for data exploration (Figure 1B and C).

## Database content and usage

### Overview of cfMethDB

**Figure 2:**
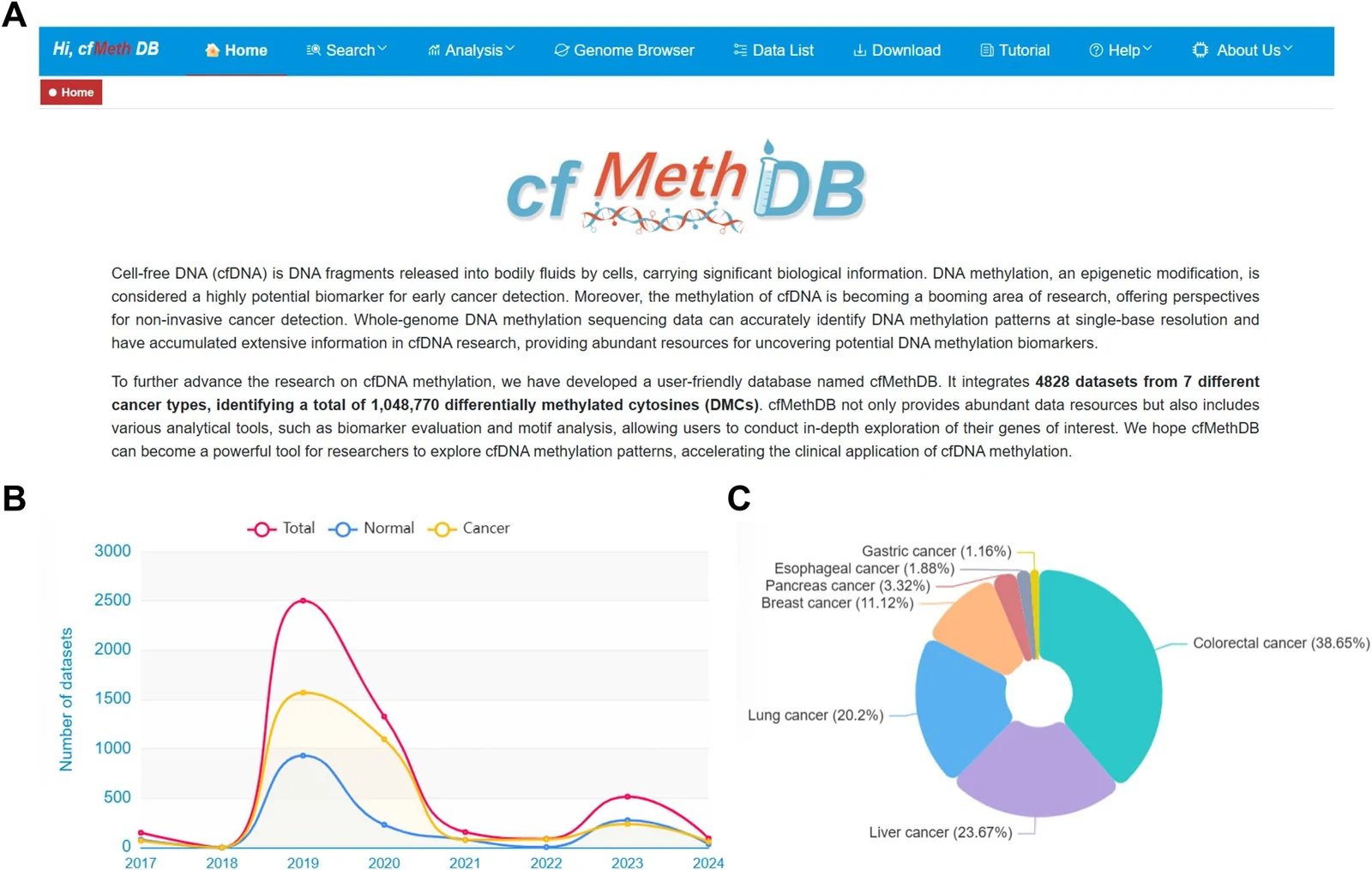
Overview of the cfMethDB database **A**. Homepage and brief introduction about cfMethDB. **B**. Statistics of cfDNA methylation datasets in cfMethDB per year. **C**. Proportions of sample groups across cfDNA methylation datasets in cfMethDB.

cfMethDB is an integrative resource for cfDNA methylation data (**Figure 2**A). The current version of cfMethDB contains 4828 datasets across 7 cancer types, with a total of 1,048,770 DMCs (Figure 2B and C; Tables S2 and S3). With a focus on the identified genome-wide DMCs, cfMethDB serves as a user-friendly platform providing a variety of functional modules (Figure 1C).

#### Search

This retrieval module allows users to explore detailed DMC information on queried genes (cfMeth Gene Search) or genomic regions (cfMeth Region Search) for each cancer type. In addition, it enables users to search for DNA methylation biomarker genes reported in the literature (Literature Search). cfMethDB also offers application programming interfaces (APIs) that allow users to conveniently query cfMeth Search results, which can be incorporated into other databases or software packages.

#### Analysis

This module includes four online tools. It provides detailed information on DMCs for a specific type of cancer (Cancer DMC Analysis), as well as across various cancer types (Pan-cancer DMC Analysis). This module can also evaluate the diagnostic value of a series of user-specified genomic regions for a particular cancer type (Marker Evaluation) and analyze the 5′-end motif patterns of genomic regions (End Motif Analysis). The data used for the Analysis module are presented in Table S4.

#### Genome Browser

This module enables users to explore DNA methylation status at single-base resolution.

#### Data List and Download

These two modules provide access to cfDNA methylation dataset information and DMC result files for browsing and downloading.

#### Tutorial, Help, and About Us

These modules provide users with detailed documentation and tutorials.

### Functional modules in cfMethDB

#### Gene Search

The “cfMeth Gene Search” module allows users to retrieve detailed DMC information for a specific cancer type. The search results include: (1) basic information about the queried gene; (2) a distribution plot of DMCs annotated to the gene; (3) detailed information on differentially methylated regions (DMRs) derived from the MethMarkerDB database [33] for the gene; and (4) gene expression information of the queried gene across different tissues from the Gene Expression Profiling Interactive Analysis (GEPIA2) database [34].

Here, we used syndecan 2 (*SDC2*) as an example to demonstrate the “cfMeth Gene Search” function and to showcase the reliability of the results within cfMethDB. The *SDC2* gene encodes a member of the heparan sulfate proteoglycan family, playing diverse roles in cell adhesion and communication [35,36]. Previous studies have demonstrated that hypermethylation of the *SDC2* promoter is a common epigenetic alteration in colorectal cancer, as observed in both tissue and serum samples [37,38]. Moreover, *SDC2* is used in an early detection kit for colorectal cancer that has been approved by the NMPA (Table S1).

**Figure 3:**
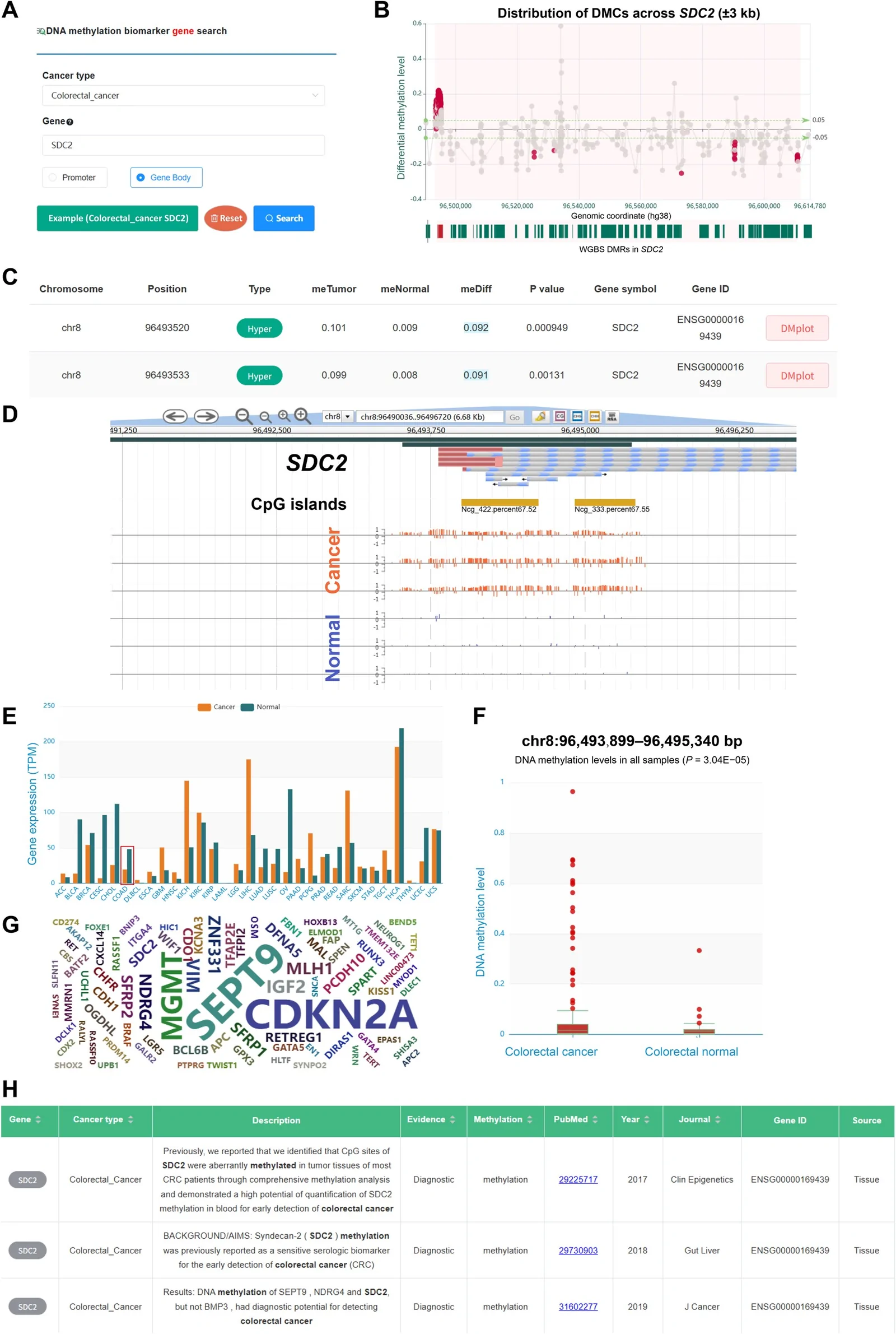
Search module in cfMethDB **A**. Screenshot of the cfMethDB Gene Search page. **B**. Distribution of DMCs across the 6-kb region centered on *SDC2* in colorectal cancer. **C**. Detailed information about the DMCs displayed in (B). **D**. Screenshot of the Genome Browser showing the DNA methylation levels around the promoter region of *SDC2* in colorectal cancer. **E**. Expression levels of *SDC2* in different types of cancer from the GEPIA2 database. COAD, one of the major types of colorectal cancer, is highlighted with a red box. **F**. DNA methylation levels of the queried *SDC2* promoter region (chr8:96,493,899–96,495,340 bp) in colorectal cancer and normal samples. **G**. Word cloud showing the previously reported biomarker genes associated with colorectal cancer. **H**. Detailed records of the reported DNA methylation biomarkers of the *SDC2* gene. WGBS, whole-genome bisulfite sequencing; DMR, differentially methylated region; *SDC2*, syndecan 2; TPM, transcripts per million; ACC, Adrenocortical Carcinoma; BLCA, Bladder Urothelial Carcinoma; BRCA, Breast Invasive Carcinoma; CESC, Cervical Squamous Cell Carcinoma and Endocervical Adenocarcinoma; CHOL, Cholangiocarcinoma; COAD, Colon Adenocarcinoma; DLBCL, Lymphoid Neoplasm Diffuse Large B-cell Lymphoma; ESCA, Esophageal Carcinoma; GBM, Glioblastoma Multiforme; HNSC, Head and Neck Squamous Cell Carcinoma; KICH, Kidney Chromophobe Renal Cell Carcinoma; KIRC, Kidney Renal Clear Cell Carcinoma; KIRP, Kidney Renal Papillary Cell Carcinoma; LAML, Acute Myeloid Leukemia; LGG, Brain Lower Grade Glioma; LIHC, Liver Hepatocellular Carcinoma; LUAD, Lung Adenocarcinoma; LUSC, Lung Squamous Cell Carcinoma; OV, Ovarian Serous Cystadenocarcinoma; PAAD, Pancreatic Adenocarcinoma; PCPG, Pheochromocytoma and Paraganglioma; PRAD, Prostate Adenocarcinoma; READ, Rectal Adenocarcinoma; SARC, Sarcoma; SKCM, Skin Cutaneous Melanoma; STAD, Stomach Adenocarcinoma; TGCT, Testicular Germ Cell Tumors; THCA, Thyroid Carcinoma; THYM, Thymoma; UCEC, Uterine Corpus Endometrial Carcinoma; UCS, Uterine Carcinosarcoma.

On the search page, users can specify the cancer type and the gene of interest, such as the *SDC2* gene in colorectal cancer (**Figure 3**A). The distribution plot of DMCs for the *SDC2* gene shows a hypermethylation region in its promoter. Detailed information on these DMCs is displayed in a table (Figure 3B and C). In addition, the Genome Browser can display single-base DNA methylation information for the hypermethylation region of the *SDC2* gene (Figure 3D). Furthermore, gene expression data reveal lower expression levels of *SDC2* in colorectal cancer samples compared with normal samples (Figure 3E), suggesting a potential correlation between promoter hypermethylation and gene silencing.

#### Region Search

Similar to the “cfMeth Gene Search” module, the “cfMeth Region Search” module allows users to search for DMCs within genomic regions of interest for a specific cancer type. The search results include detailed information about the genes and DMCs located in the queried region. Users can also access DNA methylation levels for the queried genomic region across different cancer types. For example, users can access the methylation levels of the region (chr8:96,493,899–96,495,340 bp) in colorectal cancer and normal samples (Figure 3F), enabling a direct comparison of the methylation levels between cancer and normal samples.

#### Literature Search

In the “Literature Search” module, users can select a specific cancer type, such as colorectal cancer. A word cloud is generated to display the reported DNA methylation biomarkers for that cancer type, exemplified by *SEPT9*, *CDKN2A*, and *SDC2* in colorectal cancer (Figure 3G). Moreover, users can search for a specific gene of interest, such as *SDC2*, to obtain detailed records of this biomarker in tabular format (Figure 3H).

#### Genome Browser

JBrowse is a convenient and user-friendly platform for browsing single-base DNA methylation levels. Users can select different samples to explore the DNA methylation patterns of a specific gene or genomic region. The Genome Browser reveals two CpG islands in the *SDC2* promoter that exhibit a hypermethylation pattern in colorectal cancer compared with normal samples (Figure 3D). Moreover, JBrowse includes several plugins, such as “Share” and “ScreenShot” [32]. The “Share” plugin allows users to generate a shareable URL for track information, and the “ScreenShot” plugin enables users to save the screenshots in various formats, such as PNG, JPG, and PDF. Furthermore, JBrowse supports uploading local data for browsing, including genomic repeat regions, regulatory elements, and sequencing signals in bigWig or BED formats.

#### Cancer DMC Analysis

**Figure 4:**
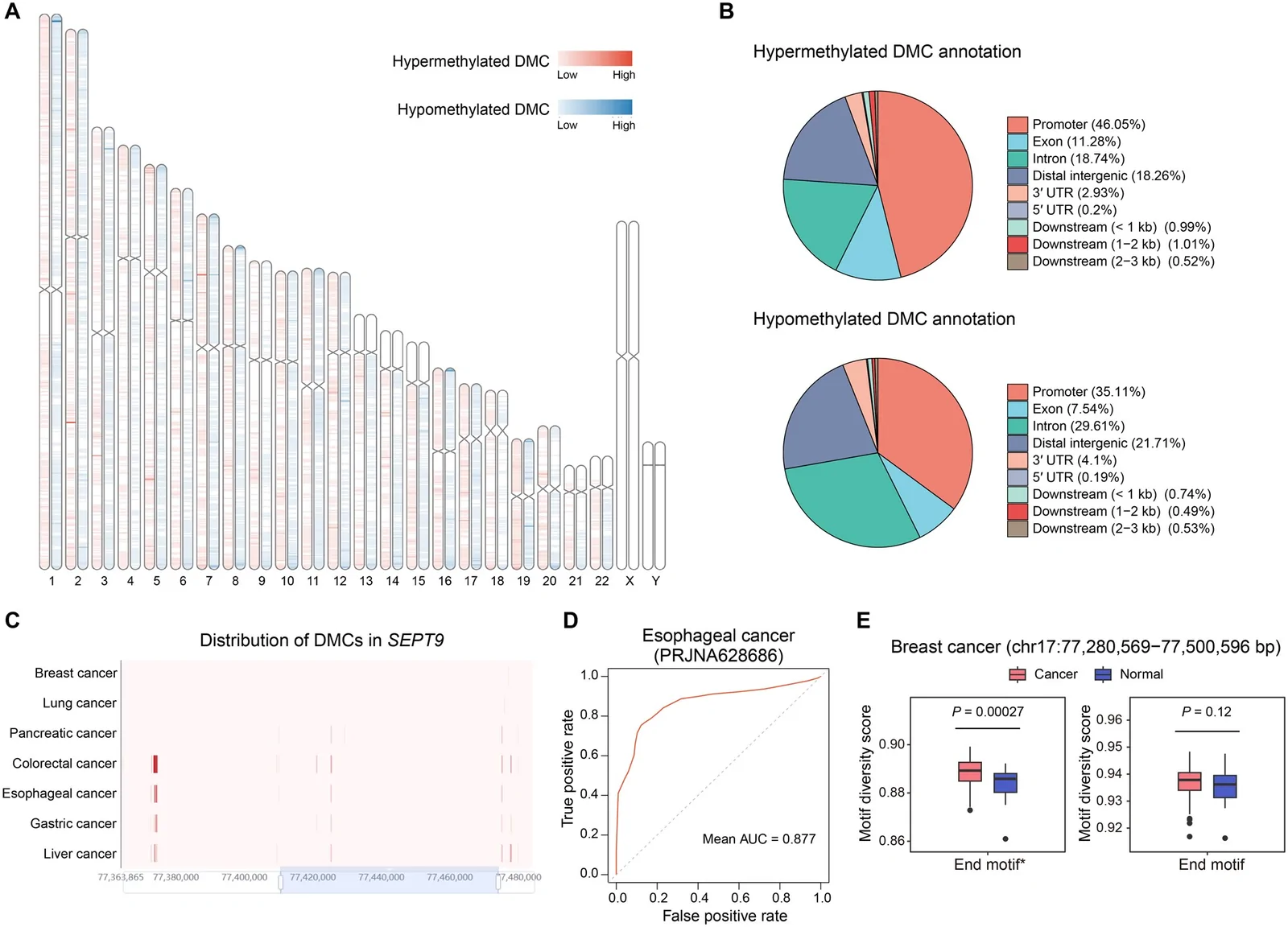
Functional modules in cfMethDB **A**. Chromosome ideogram plots displaying the density of DMCs in esophageal cancer. **B**. Genomic annotations for hypermethylated and hypomethylated DMCs in esophageal cancer. **C**. Distribution of DMCs in the *SEPT9* gene in various cancers. **D**. ROC curve showing the diagnostic performance of the *SEPT9* gene (using chr17:77,371,716–77,374,859 bp and chr17:77,428,290–77,431,433 bp as input regions) in classifying esophageal cancer and normal samples. **E**. Motif diversity score of the *SEPT9* gene in breast cancer and normal samples. “End motif*” indicates 625 end motifs with methylation status, whereas “End motif” indicates 256 end motifs without methylation status. *P* values were calculated using the Wilcoxon rank-sum test. ROC, receiver operating characteristic; AUC, area under the curve.

To better understand the distribution patterns of DMCs across the genome and compare these patterns among different cancers, we integrated the DMCs detected in various cancer types and annotated them with genomic features. In the “Cancer DMC Analysis” module, users can browse and query the results of all DMCs for various types of cancer. **Figure 4**A shows the distribution of hypermethylated and hypomethylated DMCs across each chromosome in esophageal cancer. The genomic annotation of these DMCs reveals distinct distribution patterns: hypermethylated DMCs are predominantly located in promoter regions, whereas hypomethylated DMCs show higher proportions in introns and distal intergenic regions than hypermethylated DMCs (Figure 4B). Interestingly, we identified four genomic regions characterized by a high density of hypermethylated DMCs across all cancer types (Figure S2). Notably, *HOXD* genes (chr2:176.0–176.5 Mb), *HOXA* genes (chr7:27.0–27.5 Mb), *PCDH* genes (chr5:141.0–141.5 Mb), and *ZIC1/ZIC4* genes (chr3:147.0–147.5 Mb) are located in these regions. Previous studies have reported that epigenetic dysregulation of clustered *PCDH* and *HOX* genes can serve as powerful diagnostic biomarkers [39,40]. Overall, these results suggest that cfMethDB is a valuable resource for potential biomarker discovery.

#### Pan-cancer DMC Analysis

Although several studies have investigated pan-cancer DNA methylation patterns in tissue samples [33,41,42], it remains unclear whether similar patterns exist in cfDNA. To investigate pan-cancer patterns in cfDNA, we introduced the “Pan-cancer DMC Analysis” module, which enables users to uncover potential pan-cancer methylation biomarkers. The *SIX6* and *SOX11* genes, which have been reported as promising pan-cancer biomarkers in tissue data [33,43], were also identified as promising pan-cancer biomarkers in cfDNA (Figure S3). Additionally, through cfMethDB, we revealed that hypermethylation of *SEPT9* could serve as a potential pan-cancer biomarker (Figure 4C). Collectively, the “Pan-cancer DMC Analysis” module facilitates the exploration of pan-cancer methylation patterns in cfDNA.

#### Marker Evaluation

To effectively assess the clinical application value of potential genomic regions, we developed the “Marker Evaluation” module. This module enables users to evaluate the diagnostic performance of genomic regions of interest. Recently, the *SEPT9* gene was approved by the NMPA as a biomarker for esophageal cancer detection (Table S1). As expected, our results demonstrated that the diagnostic performance of *SEPT9* in esophageal cancer is highly promising (Figure 4D). Furthermore, the “Marker Evaluation” module allows users to retrieve the DNA sequences of queried genomic regions, facilitating primer design using online tools.

#### End Motif Analysis

Recent studies have demonstrated that end motifs can reveal hallmarks of the non-random fragmentation process of cfDNA. For example, deoxyribonuclease 1L3 (*DNASE1L3*) might be linked to the generation of the CCCA end motif, whereas deoxyribonuclease 1 (*DNASE1*) might be associated with the generation of the TGTG end motif [44,45]. The diversity of end motifs suggests that these patterns can be novel biomarkers for cancer detection in the emerging field of fragmentomics [17]. Despite the importance of end motifs, there are a limited number of web servers capable of analyzing the end motifs of cfDNA. Jiang et al. demonstrated that the differential end motifs identified in whole-genome sequencing (WGS) are also detectable in WGBS, and that the motif diversity scores from WGBS and WGS are highly correlated [17]. Given that fragmentation patterns may vary across different regions of the genome [46,47], we developed the “End Motif Analysis” module to enable users to explore the end motif patterns of genes or genomic regions of interest. For example, the motif diversity score of the *SEPT9* gene was significantly higher in breast cancer samples than in normal samples (Figure 4E), indicating that there is a wide variety of plasma DNA molecules with different end motifs in plasma from breast cancer samples. Interestingly, we also observed that the difference in the motif diversity score between cancer and normal samples increased when the methylation status of the cytosine within the end motif was considered. A similar pattern was observed across the whole genome in different projects (Figure S4).

### Application of cfMethDB

**Figure 5:**
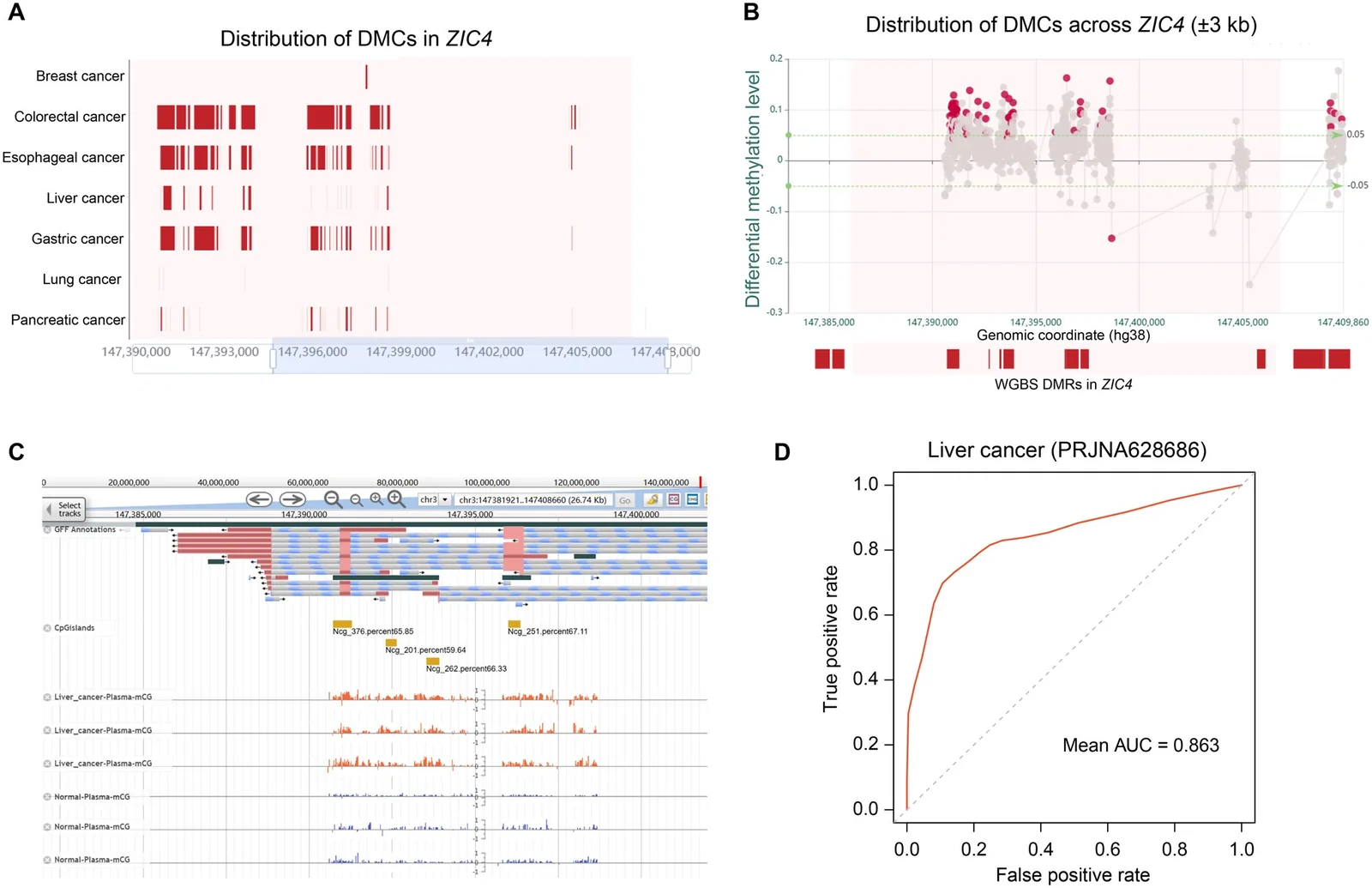
A case study in cfMethDB **A**. Distribution of DMCs in the *ZIC4* gene in various cancers. **B**. Distribution of DMCs across the 6-kb region centered on *ZIC4* in liver cancer. **C**. Screenshot of the Genome Browser showing the DNA methylation levels around the promoter region of *ZIC4* in liver cancer. **D**. ROC curve showing the diagnostic performance of the *ZIC4* gene (using chr3:147,391,246–147,391,766 bp, chr3:147,391,970–147,392,702 bp, and chr3:147,402,686–147,403,518 bp as input regions) in classifying liver cancer plasma samples and normal plasma samples.

Here, an example of how to use cfMethDB to discover potential biomarkers is presented. As described, users can identify potential biomarkers through various modules. The “Cancer DMC Analysis” module shows a high density of hypermethylated DMCs around the *ZIC1* and *ZIC4* genes. Using the “Pan-cancer DMC Analysis” module, we identified several hypermethylated regions in the *ZIC4* gene that are consistent across various cancer types in cfDNA (**Figure 5**A). To further explore this gene, we utilized the “cfMeth Gene Search” module to examine DMCs in liver cancer. The DMC distribution plot shows a high density of DMCs in the *ZIC4* gene, and the distribution pattern is similar to that of DMRs derived from tissue data (Figure 5B). Next, we used the “Genome Browser” module to display more detailed DNA methylation information for individual samples. It is evident that the *ZIC4* gene contains a hypermethylated region, particularly within the promoter for shorter transcripts (Figure 5C). Furthermore, we found that the DNA methylation level of the *ZIC4* gene can effectively distinguish liver cancer plasma samples from normal plasma samples (Figure 5D). Overall, our analysis indicates that *ZIC4* could be a potential biomarker for liver cancer diagnosis.

### Download and other features in cfMethDB

The “Download” module provides carefully curated biomarker information from published studies and identified DMCs for various cancer types. Additionally, cfMethDB allows users to download cfDNA methylation data via the “Data List” module, which enables users to search for and access samples of interest. For each sample, cfMethDB provides detailed information, including the SRA ID, sample source, PubMed ID, and bisulfite conversion rate (Figure S5A). The methylation levels and data quality of each sample, including sequencing coverage, read depth, and cfDNA fragment size distribution, are also displayed (Figure S5B–E). Furthermore, the “Contact Us” module enables users to reach out directly and submit information on data not yet in cfMethDB. We are committed to processing new submissions and regularly updating our database to incorporate the latest findings.

## Discussion

In this study, we constructed cfMethDB, a comprehensive cfDNA methylation data resource for cancer biomarkers. We collected and analyzed cfDNA methylation data from various cancer types to identify potential biomarkers across the whole genome. cfMethDB provides not only search and analysis tools for users to discover biomarkers, but also comprehensive cfDNA fragment information, including fragment length and 5′-end motif across different cancers.

In future versions, cfMethDB will be continually updated with the following improvements. (1) We will expand our data repository to encompass a wider range of cancer types. This expansion aims to enhance the comprehensiveness and applicability of our database, thereby increasing its value for both research and clinical applications. (2) We will collect single-molecule sequencing data to enrich our data resources. Single-molecule sequencing can simultaneously capture DNA sequence and DNA modification information without PCR amplification [48,49], and it can also produce longer reads than second-generation sequencing methods [50]. Yu et al. demonstrated that methylation information derived from long-read sequencing can be used to determine the tissue origin of the fetal and maternal cfDNA, with an area under the curve of 0.88 [51]. Although single-molecule sequencing data from cancer patients are currently limited, the potential of this approach should not be overlooked. In the future, we aim to incorporate single-molecule sequencing data to enhance the depth and breadth of our analysis. (3) We will introduce a multimodal analysis module. cfDNA methylation data provide not only the methylation profile but also valuable fragment information, such as the 5′-end motif and fragment size. Based on our analysis of the end motifs (Figure 4E, Figure S4), we infer that incorporating methylation status into the end motif information could enhance cancer diagnostic performance. However, a key challenge in end motif analysis lies in the inherent differences between WGS and WGBS data due to the bisulfite treatment used. Therefore, while end motif analysis in WGBS data provides valuable insights for further investigation, the results from WGS and WGBS should not be treated equivalently. In addition, we can identify genomic variants present in cfDNA. Previous studies have shown that multimodal analysis of cfDNA can improve the accuracy of cancer diagnosis [52,53]. In the future, we plan to integrate a multimodal diagnostic module into cfMethDB, with the goal of uncovering a broader spectrum of potential biomarkers through the synergistic analysis of diverse data types. (4) We will provide additional online functions based on user feedback. We are committed to keeping cfMethDB up-to-date to maintain its value as a user-friendly cfDNA methylation database. We hope that cfMethDB will facilitate the identification of cfDNA methylation biomarkers and contribute to the advancement of their clinical applications.

## Supplementary Material

qzaf092_Supplementary_Data

## Data Availability

The cfMethDB database is available at https://cfmethdb.hzau.edu.cn/home, and it can be accessed without registration or login. cfMethDB has been submitted to Database Commons [54] at the National Genomics Data Center (NGDC), China National Center for Bioinformation (CNCB), which is publicly accessible at https://ngdc.cncb.ac.cn/databasecommons/database/id/9685.

## CRediT author statement

**Yuanhui Sun:** Data curation, Formal analysis, Visualization, Writing – original draft, Writing – review & editing. **Zhixian Zhu:** Data curation, Formal analysis, Writing – review & editing. **Qiangwei Zhou:** Formal analysis, Visualization, Writing – review & editing. **Zhe Wang:** Data curation. **Yuying Hou:** Data curation. **Xionghui Zhou:** Writing – review & editing. **Guoliang Li:** Conceptualization, Supervision, Writing – review & editing, Funding acquisition. All authors have read and approved the final manuscript.
