# Supplementary material for: cfMethDB: A Comprehensive cfDNA Methylation Data Resource for Cancer Biomarkers
Source: Genomics Proteomics Bioinformatics. 2025 Oct 14;24(2):qzaf092. doi: 10.1093/gpbjnl/qzaf092 (PMC13544964; doi:10.1093/gpbjnl/qzaf092)
Supplement: Supplementary file 1 [file qzaf092_supplementary_data.zip › Figure S1.pdf]

Figure S1

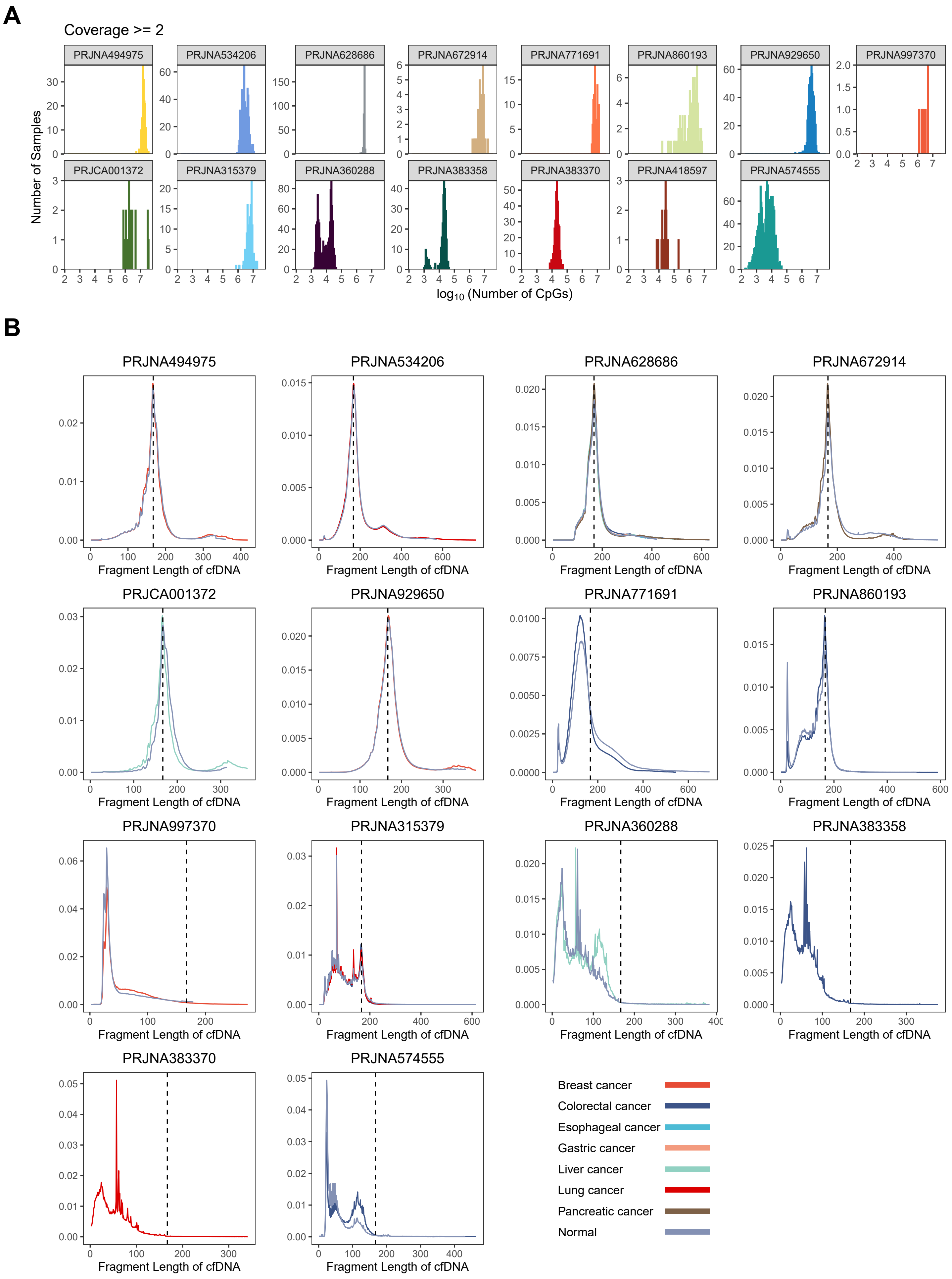
A
Coverage >= 2
PRJNA494975
PRJNA534206
PRJNA628686
PRJNA672914
PRJNA771691
PRJNA860193
PRJNA929650
PRJNA997370
PRJCA001372
PRJNA315379
PRJNA360288
PRJNA383358
PRJNA383370
PRJNA418597
PRJNA574555
Number of Samples
log10 (Number of CpGs)
B
Fragment Length of cfDNA
Breast cancer
Colorectal cancer
Esophageal cancer
Gastric cancer
Liver cancer
Lung cancer
Pancreatic cancer
Normal
