# Supplementary material for: cfMethDB: A Comprehensive cfDNA Methylation Data Resource for Cancer Biomarkers
Source: Genomics Proteomics Bioinformatics. 2025 Oct 14;24(2):qzaf092. doi: 10.1093/gpbjnl/qzaf092 (PMC13544964; doi:10.1093/gpbjnl/qzaf092)
Supplement: Supplementary file 1 [file qzaf092_supplementary_data.zip › Figure S2.pdf]

Figure S2

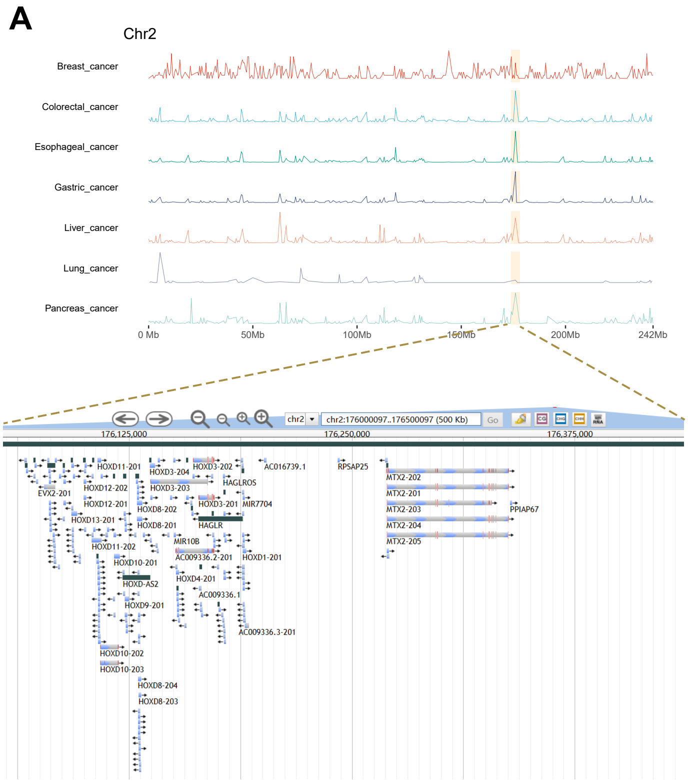

HOXD Genes (chr2 : 176 Mb - 176.5 Mb)

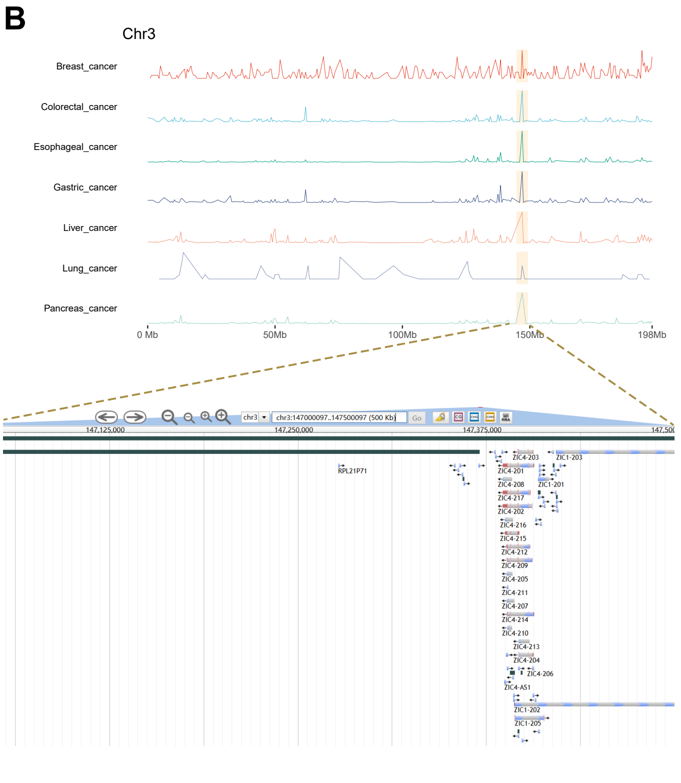

ZIC1/ZIC4 Genes (chr3 : 147 Mb - 147.5 Mb)

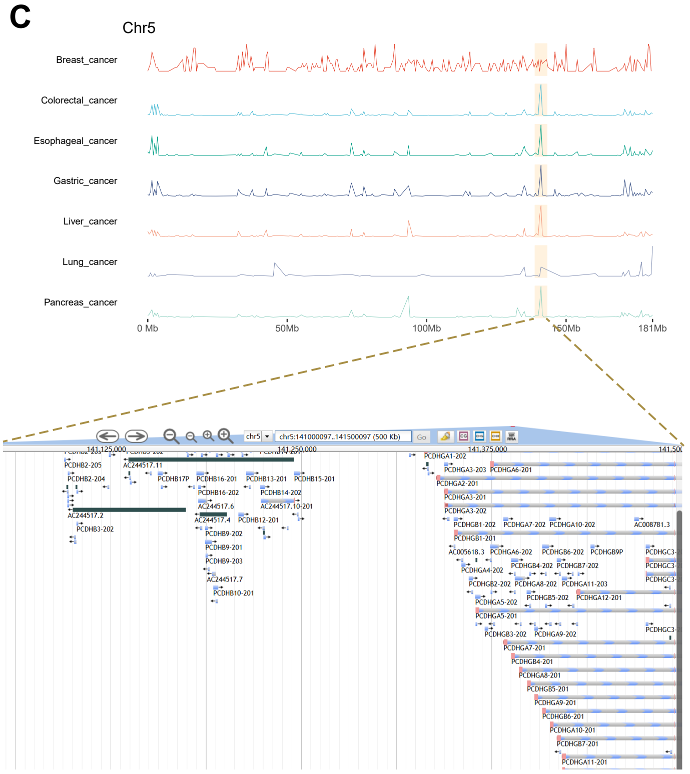

PCDH Genes (chr5 : 141 Mb - 141.5 Mb)

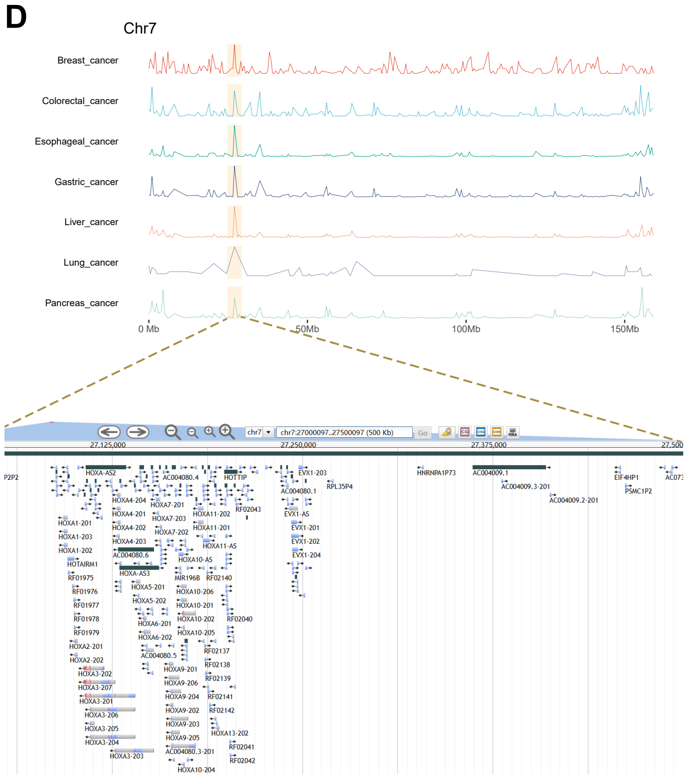

HOXA Genes (chr7 : 27 Mb - 27.5 Mb)
