# Supplementary material for: cfMethDB: A Comprehensive cfDNA Methylation Data Resource for Cancer Biomarkers
Source: Genomics Proteomics Bioinformatics. 2025 Oct 14;24(2):qzaf092. doi: 10.1093/gpbjnl/qzaf092 (PMC13544964; doi:10.1093/gpbjnl/qzaf092)
Supplement: Supplementary file 1 [file qzaf092_supplementary_data.zip › Figure S3.pdf]

Figure S3

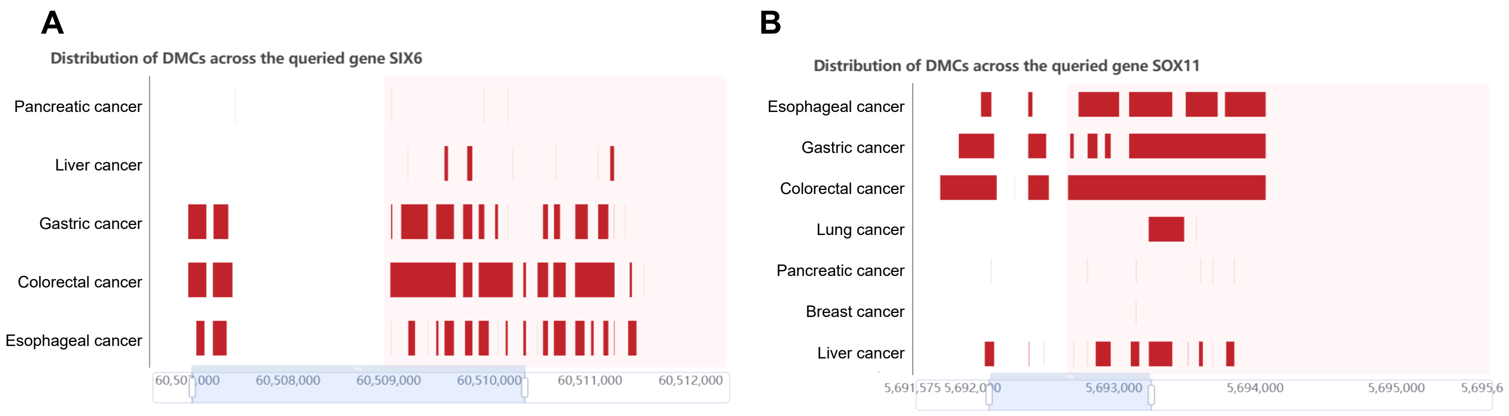
A
Distribution of DMCs across the queried gene SIX6
Pancreatic cancer
Liver cancer
Gastric cancer
Colorectal cancer
Esophageal cancer
60,508,000
60,509,000
60,510,000
60,511,000
60,512,000
B
Distribution of DMCs across the queried gene SOX11
Esophageal cancer
Gastric cancer
Colorectal cancer
Lung cancer
Pancreatic cancer
Breast cancer
Liver cancer
5,691,575
5,693,000
5,694,000
5,695,000
