# Supplementary material for: cfMethDB: A Comprehensive cfDNA Methylation Data Resource for Cancer Biomarkers
Source: Genomics Proteomics Bioinformatics. 2025 Oct 14;24(2):qzaf092. doi: 10.1093/gpbjnl/qzaf092 (PMC13544964; doi:10.1093/gpbjnl/qzaf092)
Supplement: Supplementary file 1 [file qzaf092_supplementary_data.zip › Figure S4.pdf]

Figure S4

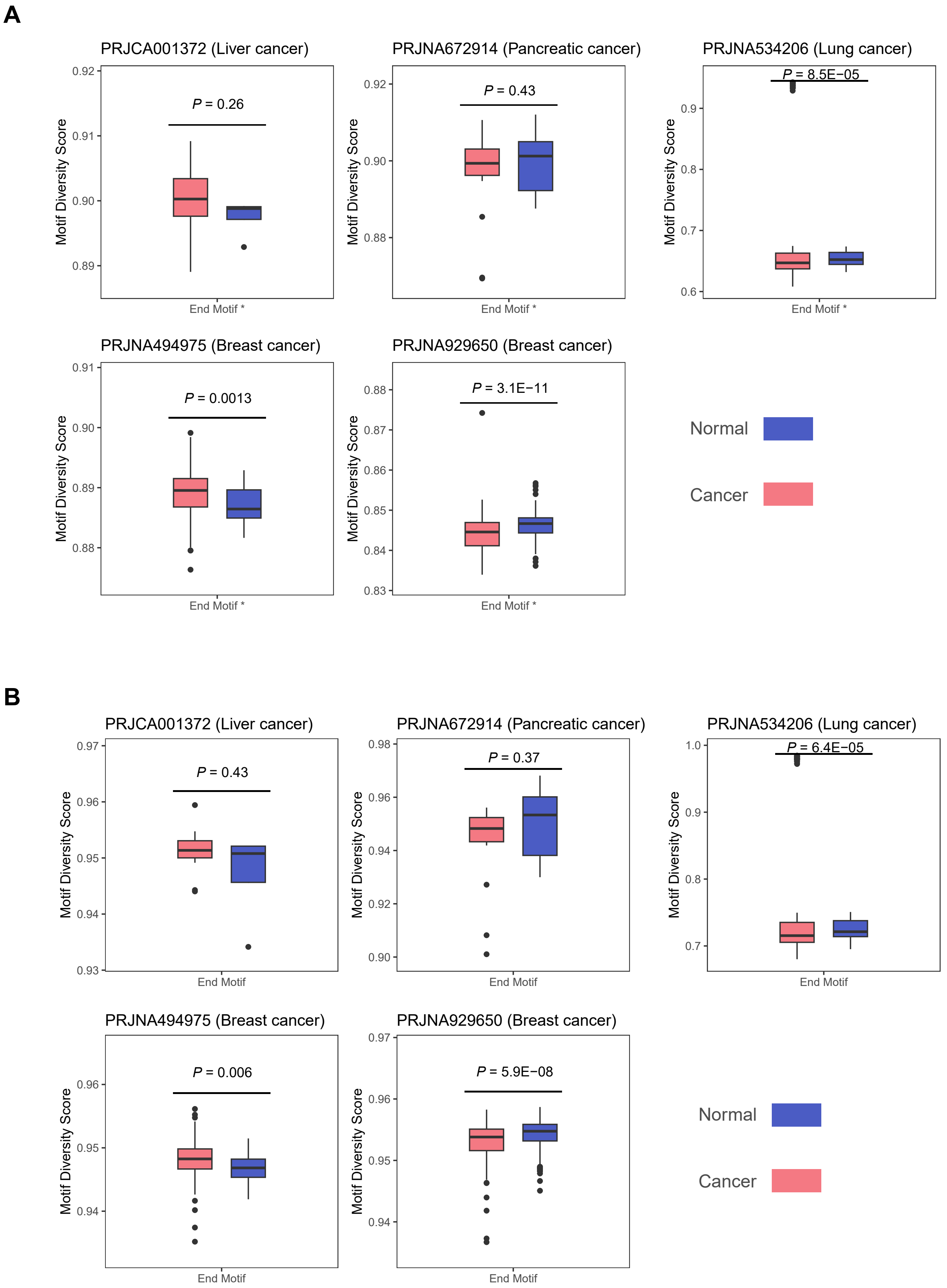
A
PRJCA001372 (Liver cancer)
P = 0.26
Motif Diversity Score
End Motif *
PRJNA672914 (Pancreatic cancer)
P = 0.43
Motif Diversity Score
End Motif *
PRJNA534206 (Lung cancer)
P = 8.5E−05
Motif Diversity Score
End Motif *
PRJNA494975 (Breast cancer)
P = 0.0013
Motif Diversity Score
End Motif *
PRJNA929650 (Breast cancer)
P = 3.1E−11
Motif Diversity Score
End Motif *
Normal
Cancer
B
PRJCA001372 (Liver cancer)
P = 0.43
Motif Diversity Score
End Motif
PRJNA672914 (Pancreatic cancer)
P = 0.37
Motif Diversity Score
End Motif
PRJNA534206 (Lung cancer)
P = 6.4E−05
Motif Diversity Score
End Motif
PRJNA494975 (Breast cancer)
P = 0.006
Motif Diversity Score
End Motif
PRJNA929650 (Breast cancer)
P = 5.9E−08
Motif Diversity Score
End Motif
Normal
Cancer
